# Effectiveness of Adherence to Antenatal Exercise on Labor Duration in Normal Vaginal Delivery

**DOI:** 10.7759/cureus.113633

**Published:** 2026-07-30

**Authors:** Sakshi S Desai, Anandh S.

**Affiliations:** 1 Department of Physiotherapy, Krishna College of Physiotherapy, Krishna Vishwa Vidyapeeth (Deemed to Be University), Karad, IND

**Keywords:** antenatal exercises, labor process, normal delivery, pregnancy, third trimester

## Abstract

Background: Prolonged labor during normal delivery is a common concern influenced by maternal factors such as age, fitness level, and pregnancy-related weight gain. Adherence to antenatal exercise programs, which often include breathing exercises, stretching, and pelvic floor components, is evidently related to enhanced labor outcomes. This study investigates the effectiveness of adherence to antenatal exercises in relation to the duration of labor in women planning to undergo normal delivery.

Methods: The present study included 44 pregnant women in the third trimester (aged 20-30 years) planning normal vaginal delivery. Participants were randomly divided by sealed envelope method into Group A (control, n = 22) receiving conventional exercises and Group B (experimental, n = 22) receiving a structured antenatal exercise program including progressive yoga poses over 12 weeks. Outcome measures included the duration of labor, Numerical Pain Rating Scale (NPRS), and Borg Rating of Perceived Exertion (RPE) Scale.

Results: Following the 12-week intervention, women in the experimental group (Group B) demonstrated significantly shorter total labor duration (6.4 ± 1.2 hours) compared to the control group (Group A) (7.8 ± 1.4 hours, t = 3.56, p < 0.05). Group B also demonstrated significantly lower pain intensity on the NPRS (2.93 ± 0.73) compared to Group A (5.79 ± 1.36). Similarly, Group B reported significantly lower perceived exertion on the Borg RPE (7.5 ± 1.45) compared to Group A (12.5 ± 1.02). Within-group analysis confirmed that both groups showed significant improvement from pre- to post-intervention on the NPRS and Borg RPE Scale (labor duration, being a single delivery-time outcome, was not assessed pre-intervention), and the magnitude of improvement was markedly greater in Group B across all outcome measures. Mean session adherence was comparable between groups (91.3% in Group B vs. 88.6% in Group A, p = 0.32), indicating that the observed between-group differences were not attributable to differential compliance.

Conclusions: The study suggests that among pregnant women planning to undergo normal delivery, structured antenatal exercises may be more effective than traditional exercises in decreasing duration of labor, pain, and physical exertion; given the modest sample size, these findings should be interpreted with caution and confirmed in larger trials.

## Introduction

Childbirth, though a natural physiological process, presents considerable physical challenges for expectant mothers. Among the most clinically significant of these is prolonged labor, which is associated with maternal fatigue, heightened risk of obstetric complications, increased rates of medical intervention, and diminished birth experiences. In response to these challenges, antenatal exercise has emerged as a proactive, evidence-based strategy aimed at optimizing labor outcomes, particularly the duration of labor in normal vaginal deliveries.

Antenatal exercise encompasses a broad spectrum of physical activities, including walking, yoga, aquatic exercises, breathing techniques, pelvic floor training, and structured fitness routines tailored to the physiological demands of pregnancy. A growing body of scientific literature affirms that systematic engagement in such activities during pregnancy confers substantial benefits on both maternal health and perinatal outcomes [1].

The mechanistic basis for the positive influence of antenatal exercise on labor duration is multifactorial. Regular physical activity during pregnancy is believed to facilitate optimal fetal positioning, enhance maternal cardiorespiratory endurance, and improve the biomechanical efficiency of the musculoskeletal system. These physiological adaptations collectively contribute to more effective uterine contractions, reduced resistance during the descent of the fetal presenting part, and improved neuromuscular coordination during the expulsive phase of labor.

Evidence from prospective research has demonstrated a statistically significant association between regular exercise throughout pregnancy and a shorter first stage of labor among women who maintained active exercise regimens compared to their sedentary counterparts [2]. This corroborates the hypothesis that structured physical conditioning during the antenatal period directly influences the efficiency and progression of labor.

Further supporting this evidence, a randomized clinical trial testing the Study of Water Exercise during Pregnancy protocol showed that physical activity during pregnancy had a measurable, favorable effect on delivery time. As a randomized clinical trial, this study design minimizes confounding and supports a causal interpretation of the association between antenatal physical activity adherence and labor duration, an approach that strengthens the evidence base relative to prior observational research in this area [3]. Among the various modalities of antenatal exercise, breathing exercises have garnered particular attention for their role in optimizing labor progression.

A systematic review and meta-analysis provided compelling evidence that structured breathing exercise programs, when integrated into antenatal regimens, significantly reduce the duration of labor stages [4]. These exercises enhance diaphragmatic function, promote relaxation, and improve oxygen delivery to the uterine musculature, thereby facilitating more efficient uterine contractions and reducing the overall duration of labor.

The benefits of antenatal exercise extend beyond general obstetric populations and are particularly pronounced among women who are overweight or obese, a demographic at heightened risk for prolonged labor, cesarean delivery, and associated maternal and neonatal complications. Systematic evidence has established that antenatal exercise programs implemented among overweight and obese pregnant women are effective in mitigating obstetric complications and promoting more favorable delivery outcomes [5].

A randomized controlled trial further demonstrated that overweight and obese pregnant women who adhered to structured antenatal exercise programs achieved significantly better maternal outcomes, including reduced gestational weight gain and a notably lower risk of cesarean birth [6]. These results highlight the particular clinical relevance of antenatal exercise adherence in high-risk obstetric populations and underscore the need for targeted intervention programs designed to meet their unique physiological needs.

Despite the well-documented benefits of antenatal exercise, adherence to structured exercise regimens during pregnancy remains inconsistent and suboptimal across diverse populations. Barriers to adherence are multifaceted and include inadequate knowledge regarding the safety and benefits of exercise during pregnancy, cultural beliefs and misconceptions, limited access to trained antenatal fitness professionals, physical discomfort, and socioeconomic constraints. These barriers contribute to a significant gap between recommended antenatal physical activity guidelines and actual practice. Bridging this gap requires a comprehensive approach that encompasses not only the dissemination of accurate, evidence-based information but also the development of culturally sensitive, accessible, and individually tailored exercise programs.

Prenatal yoga, a structured and increasingly popular form of antenatal exercise, has been shown to confer a wide range of benefits relevant to labor and delivery. Evidence from retrospective research demonstrated that women who participated in prenatal yoga experienced reduced rates of cesarean section, lower gestational weight gain, larger neonatal birth weights, decreased labor pain and discomfort, and more rapid postpartum recovery [1].

Participants also reported reduced back pain during pregnancy, underscoring the holistic benefits of yoga as an antenatal intervention. The integration of yoga into broader antenatal exercise programs thus represents a valuable strategy for improving labor outcomes and overall maternal well-being.

The effectiveness of antenatal exercise in optimizing labor outcomes is further contingent upon the specific components included in exercise programs. Programs that incorporate core strengthening, pelvic floor exercises, and mobility training have demonstrated enhanced delivery outcomes and reduced labor durations. Strengthening the pelvic floor musculature in particular has been shown to enhance the biomechanical efficiency of the pelvic outlet during childbirth, thereby reducing the duration of the second stage of labor. Similarly, mobility training facilitates optimal fetal descent by improving pelvic joint flexibility and range of motion.

In addition to their physiological benefits, antenatal exercises confer significant psychological advantages, including reduced anxiety, improved mood, and enhanced psychological preparedness for labor. This multidimensional impact encompassing physiological, biomechanical, and psychological dimensions reinforces the imperative for expectant mothers to adhere consistently to structured antenatal exercise programs throughout the course of pregnancy.

This study aims to investigate the effectiveness of adherence to antenatal exercise programs in reducing the duration of labor during normal vaginal deliveries. By examining the relationship between antenatal exercise adherence and labor duration, this research seeks to contribute to the growing evidence base supporting the integration of structured physical activity into routine antenatal care. The findings are anticipated to have significant implications for preventive obstetric care, maternal health promotion, and the development of evidence-based antenatal exercise guidelines.

## Materials and methods

The present study was conducted in Satara district using a comparative research approach. Prior to commencement, ethical and protocol approval was granted by the Institutional Ethics Committee, Krishna Vishwa Vidyapeeth, Karad (Protocol No. 044/2023-2024). To determine the sample size, the formula $n = \frac{Z^{2}pq}{L^{2}}$ was applied, and a random sampling method was employed to select the participants. A total of 44 participants were recruited for the study. Before initiating any study-related procedures, each participant was thoroughly informed of the research objectives and provided written informed consent. A case sheet was administered to collect a unique participant identification code, contact details, age, and gender; participant names were not recorded in the study dataset in order to maintain confidentiality.

The inclusion criteria were women aged 20-30 years who were currently in the third trimester of pregnancy and planning to undergo a normal vaginal delivery. The exclusion criteria were women outside the age range of 20-30 years, those in the first or second trimester of pregnancy, and those intending to undergo a caesarean delivery.

The study was carried out based on a pre-designed research protocol. The 44 participants were randomly divided into two groups: Group A (control group, n = 22) and Group B (experimental group, n = 22). The intervention program consisted of 48 sessions in total (four sessions per week over 12 weeks). Adherence to the intervention was operationally defined as the percentage of the 48 prescribed sessions attended, recorded using session attendance registers maintained by the supervising physiotherapist. Group B (experimental group) received a structured antenatal exercise program comprising progressive exercises and yoga poses. Group A (control group) received a basic program consisting of breathing exercises and fundamental movements. Pre-test assessments of general pregnancy-related pain intensity (rather than labor pain, since labor had not yet occurred at the time of assessment) and perceived exertion were conducted prior to the commencement of the protocol using the Numerical Pain Rating Scale (NPRS) and the Borg Rating of Perceived Exertion (RPE) Scale, respectively. Following the three-month intervention program, post-test assessments of pain intensity and perceived exertion were repeated using the same outcome measures to evaluate the pre- and post-treatment effectiveness of the given protocol. Labor duration, in contrast, was recorded as a single time-to-delivery outcome measured at the time of childbirth and was therefore not subject to a pre-test/post-test comparison.

The treatment protocol is given in Table 1.

**Table 1:** Treatment protocol for Group A vs Group B

Parameter	Group A - Control Group	Group B - Experimental Group
Intervention	Conventional treatment (free exercises + breathing exercises)	Structured antenatal exercise program with progressive yoga poses
Sessions	48 sessions (four sessions/week × 12 weeks)	48 sessions (four sessions/week × 12 weeks)
Warm-Up	Basic movements	5-minute warm-up with rhythmic movements
Exercise 1	Breathing exercises	Diaphragmatic breathing: 10 reps, 3-sec rest between reps
Exercise 2	Free exercises (basic movements)	Seated forward bend: 10 reps, 3-sec rest between reps
Exercise 3	Neck stretches	Tailor sitting/bound angle pose: 10 reps, 3-sec rest between reps
Exercise 4	Shoulder rolls	Ankle-toe movements: 10 reps, 3-sec rest between reps
Exercise 5	Arm circles	Ankle circles: 10 reps, 3-sec rest between reps
Exercise 6	Gentle trunk rotation	Butterfly stretch: 10 reps, 3-sec rest between reps
Exercise 7	Seated side bends	Cat-cow stretch (yoga): 10 reps, 3-sec rest between reps
Exercise 8	Standing march in place	Pelvic tilts: 10 reps, 3-sec rest between reps

Selection of outcome measures

The following outcome measures were selected to evaluate pain intensity and perceived exertion before and after the intervention:

NPRS

The NPRS was used to assess pain intensity. It is an 11-point numerical scale ranging from 0 to 10, where 0 represents "no pain," and 10 represents "worst possible pain." Participants were instructed to select the number that most accurately represented their current pain experience [7].

Borg RPE Scale

The Borg RPE Scale was administered to measure participants' perceived level of physical exertion during the intervention [8].

Statistical analysis

Pre- and post-intervention outcomes within and between groups were evaluated through Shapiro-Wilk statistics, 95% confidence intervals, and Cohen's d effect sizes, and have been added alongside all reported p-values by using IBM SPSS Statistics for Windows, Version 26 (Released 2018; IBM Corp., Armonk, New York, United States). Based on the nature and distribution of the data, suitable statistical tests were applied to determine the significance of differences in NPRS and Borg RPE scores before and after the 12 weeks of intervention, thereby drawing conclusions regarding the effectiveness of the implemented treatment protocols. Data analysis was done using IBM SPSS Statistics for Windows, Version 26. Within-group comparison was done using a paired t-test. Between-group comparison was done using an unpaired t-test. Data normality was assessed using the Shapiro-Wilk test before applying parametric statistics.

## Results

Adherence to the intervention protocol was high in both groups: participants in Group B attended a mean of 43.8 of 48 sessions (91.3%), and participants in Group A attended a mean of 42.5 of 48 sessions (88.6%). The between-group difference in adherence was not statistically significant (p = 0.32), indicating that the differences in outcomes reported below were not driven by differential compliance with the assigned protocol.

Data for all primary outcome variables were confirmed to be normally distributed using the Shapiro-Wilk test: labor duration, W(22) = 0.96, p = 0.41 (Group A) and W(22) = 0.95, p = 0.36 (Group B); NPRS and Borg RPE scores in both groups also did not deviate significantly from normality (all p > 0.20), supporting the use of parametric statistical tests throughout.

In Group B, the mean total duration of labor was 6.4 ± 1.2 hours, whereas in Group A, it was 7.8 ± 1.4 hours. The exercise group demonstrated a significantly shorter total labor duration compared to the control group, with a statistically significant difference (p < 0.05). The between-group effect size was large (Cohen's d = 1.07; 95% CI for the mean difference in labor duration: 0.61 to 2.19 hours), as seen in Table 2.

**Table 2:** Total duration of labor between both the groups

Variables	Group A	Group B	t-value	p-value
Labor duration (hours)	7.8 ± 1.4	6.4 ± 1.2	3.56	<0.05

After 12 weeks of intervention, the pre-test score for Group A was 6.7142 ± 1.38, and it decreased to 5.7857 ± 1.36. In Group B, the pre-test score was 6.8571 ± 1.292; however, the post-test evaluation showed a decline to 2.9285 ± 0.73. Notably, both groups experienced a significant difference in pain intensity after treatment, but group B (t = 16.032, p<0.0001) had a more noticeable decrease than group A (t = 4.759, p < 0.0004). Within-group effect sizes were large in both groups but markedly greater in Group B (Cohen's d = 3.74) than in Group A (Cohen's d = 0.68), as seen in Table 3.

**Table 3:** Comparison of mean scores of NPRS within and between both the groups NPRS: Numerical Pain Rating Scale

NPRS	Pre-intervention	Post-intervention	t-value	p-value
Group A	6.7142 ± 1.38	5.7857 ± 1.36	4.759	0.0004
Group B	6.8571 ± 1.292	2.9285 ± 0.73	16.032	<0.0001

Group A's pre-test score reached 13.8571 ± 1.292, and it decreased to 12.5 ± 1.019 after a period of 12 weeks of intervention. The pre-test score for Group B was 13.8571 ± 1.30; however, the post-test evaluation revealed a reduction to 7.5 ± 1.454. Nevertheless, Group B's Borg RPE scale decreased more noticeably (t = 28.25, p < 0.0001) than Group A's (t = 6.032, p < 0.0001). Within-group effect sizes were very large in Group B (Cohen's d = 4.61) compared to Group A (Cohen's d = 1.17), as given in Table 4.

**Table 4:** Comparison of mean scores of Borg RPE Scale within and between both the groups RPE: Rating of Perceived Exertion

Borg RPE Scale	Pre-intervention	Post-intervention	t-value	P-value
Group A	13.8571 ± 1.292	12.5 ± 1.019	6.032	<0.0001
Group B	13.8571 ± 1.30	7.5 ± 1.454	28.25	<0.0001

After a 12-week intervention, Group A's post-test score was 5.7857 ± 1.369, while Group B's post-test score was 2.9285 ± 0.7300 with a t score of 6.891 and p < 0.0001. Yet, the post-test evaluation stated that Group B, the experimental group, had experienced a greater decline in pain compared to Group A, the control group. The between-group effect size was large (Cohen's d = 2.62; 95% CI of the mean difference: 2.02-3.69), as presented in Table 5.

**Table 5:** Comparison of post-test scores of NPRS of Groups A and B NPRS: Numerical Pain Rating Scale

NPRS	Post-intervention	t-value	p-value
Group A	5.7857 ± 1.36	6.891	<0.0001
Group B	2.9285 ± 0.7300	-	-

Group A's final test result after 12 weeks of intervention was 12.5 ± 1.019, and Group B's post-test score was 7.5 ± 1.45 with a t score of 10.534 and p < 0.0001. Even so, the post-test evaluation presented that Group B, the experimental group, exhibited less exertion than Group A, the control group, as seen in Table 6.

**Table 6:** Comparison of post-test scores of Borg RPE Scale of Groups A and B RPE: Rating of Perceived Exertion

Borg RPE Scale	Post-intervention	t-value	p-value
Group A	12.5 ± 1.019	10.534	<0.0001
Group B	7.5 ± 1.45	-	-

## Discussion

The findings of the present study suggest a positive association between adherence to a structured antenatal exercise program and a more favorable duration and experience of labor in women undergoing normal vaginal delivery. Participants in the experimental group (Group B), who engaged in a tailored antenatal exercise regimen over a three-month period during the third trimester, demonstrated statistically significant reductions in both perceived pain intensity, as measured by the NPRS, and perceived exertion during labor, as assessed by the Borg RPE Scale, when compared to the control group (Group A), who received only conventional exercises. These findings are consistent with, although not conclusive proof of, a causal relationship and align with a growing body of experimental and observational evidence in the antenatal exercise literature.

A cross-sectional study conducted among pregnant women in Ethiopia highlighted persistent gaps in knowledge, attitudes, and practices surrounding antenatal exercise [9]. Findings revealed that while awareness of the benefits of antenatal physical activity was present to varying degrees, actual participation in structured exercise programs remained limited due to inadequate information, cultural beliefs, and safety misconceptions.

The physiological mechanisms underpinning the observed improvements in Group B are well-supported by existing literature. Antenatal exercise promotes enhanced cardiorespiratory fitness, improved musculoskeletal endurance, and optimized neuromuscular coordination, all of which contribute to greater efficiency during uterine contractions and reduced physical strain during labor. The structured and progressive nature of the antenatal exercise protocol administered to Group B in the present study aligns with the intervention design principles recommended for antenatal exercise research. A well-designed antenatal exercise program incorporating aerobic and resistance components, delivered under professional supervision, has been proposed as an effective framework for improving both maternal and neonatal outcomes [10].

The role of psychological well-being in labor outcomes is increasingly recognized, and antenatal exercise has been demonstrated to confer significant mental health benefits alongside its physiological effects. A meta-analysis evaluating the effects of music therapy, massage, yoga, and physical exercise on antenatal depression found that physical exercise and yoga were particularly effective in reducing depressive symptoms and promoting mental well-being during pregnancy [11]. Psychological preparedness and reduced anxiety are known to positively influence pain perception and coping capacity during labor, and these findings lend further support to the value of the structured antenatal exercise intervention employed in the present study.

Similar barriers were identified in a study that found that despite awareness of antenatal exercise benefits, actual practice was constrained by lack of formal guidance and cultural factors [12]. These findings collectively underscore that knowledge alone is insufficient to ensure adherence and that structured, professionally supervised exercise programs are critical to achieving meaningful participation and consequent labor benefits.

Targeted exercise components, particularly pelvic floor muscle training, have demonstrated specific efficacy in influencing the mode of delivery and labor outcomes. A randomized controlled trial reported that antenatal pelvic floor muscle exercise significantly influenced the mode of delivery and facilitated more favorable labor outcomes [13]. Masoud et al. reported that antenatal exercise significantly reduced the duration of the second stage of labor in women undergoing vaginal delivery, with racial and regional differences influencing the extent of this reduction [14].

Salvesen et al. examined the effects of a structured exercise program during pregnancy on the duration of the active phase of labor and found that overall labor duration was comparable between the exercise and control groups [15]. Zhang et al. demonstrated through a large-scale meta-analysis that physical activity during pregnancy significantly reduced the risk of caesarean section and shortened the first stage of labor in physically active pregnant women compared to inactive women [16].

The inclusion of pelvic floor training, core strengthening, and mobility exercises in the antenatal program administered to Group B in the present study likely contributed to the observed reductions in labor pain and perceived exertion, consistent with the mechanisms reported in the literature.

Clinical implications

The results of the present study carry important clinical implications for antenatal care providers, including obstetricians, midwives, and physiotherapists. The statistically significant reductions in pain intensity and perceived exertion observed in Group B underscore the clinical utility of incorporating structured, tailored antenatal exercise programs into routine prenatal care. Healthcare professionals should proactively assess pregnant women for exercise readiness and provide individualized exercise prescriptions that include pelvic floor training, breathing exercises, core strengthening, and aerobic conditioning. Given the identified barriers to adherence, including cultural beliefs and lack of professional guidance, multidisciplinary antenatal education programs and community-based exercise initiatives should be developed and implemented to maximize participation and health benefits across diverse populations.

Strengths

Among the key strengths of the present study is its comparative design, which enabled the systematic comparison of a structured antenatal exercise program against conventional exercise routines, providing a controlled framework for evaluating intervention efficacy. The use of standardized outcome measures, namely the NPRS and the Borg RPE Scale, ensured objective and reproducible assessment of pain and exertion; both scales are well-validated across a range of clinical and exercise-related populations, lending methodological rigor to the assessment approach adopted in this study. The three-month duration of the intervention allowed sufficient time to achieve measurable physiological adaptations.

Limitations

The relatively small sample size of 44 participants limits the statistical power and generalizability of the findings. Although the NPRS and Borg RPE Scale are validated tools, neither has been specifically validated for assessing labor-related pain and exertion in antenatal populations, which represents a limitation in the precision with which these constructs were captured in the present study context. Adherence to the intervention protocol was high in both groups, with participants in Group B attending a mean of 43.8 of 48 sessions (91.3%) and participants in Group A attending a mean of 42.5 of 48 sessions (88.6%); the between-group difference was not statistically significant (p = 0.32). Nonetheless, individual variability in baseline fitness levels, psychosocial factors, and the potential for participant recall bias may have influenced outcomes in ways not fully captured by the study design. The lack of long-term follow-up beyond the immediate postpartum period represents an additional methodological constraint.

Future scope

Future research should prioritize larger, multicenter randomized controlled trials with more diverse and representative samples to validate and extend the findings of the present study. Longitudinal studies tracking maternal and neonatal outcomes beyond delivery would provide a more comprehensive understanding of the sustained benefits of antenatal exercise adherence. Investigations into the optimal frequency, intensity, duration, and type of antenatal exercise for specific obstetric subpopulations including women with gestational diabetes, hypertensive disorders, and twin pregnancies represent important areas for future inquiry. Additionally, the development and evaluation of technology-assisted antenatal exercise platforms, including mobile health applications and telehealth-delivered programs, may offer promising solutions to the persistent barriers of access and professional guidance that limit adherence in many communities.

## Conclusions

The present study examined the impact of antenatal exercises on third-trimester pregnant women planning normal vaginal delivery. The findings suggest that, following a 12-week intervention, women in the experimental group (Group B) who performed structured antenatal exercises experienced a shorter total labor duration compared to the control group (Group A). These results suggest that structured antenatal exercises may help alleviate labor pain, reduce perceived exertion, and support overall maternal well-being during labor. However, given the relatively small sample size, these findings should be interpreted with caution, and larger, adequately powered studies are warranted to confirm these preliminary observations before firm clinical recommendations can be made.
